# Association between physical function parameters and fracture risk in older women in primary health care in southern Brazil

**DOI:** 10.1007/s11657-026-01688-8

**Published:** 2026-03-19

**Authors:** Léo Canterle Dal Osto, Luana Fioravanti Roland, Mariá Nunes Pinto, Vitor Pelegrim de Oliveira, Kawoana Trautman Vianna, Renato Gorga Bandeira de Mello, Poli Mara Spritzer, Tayane Muniz Fighera

**Affiliations:** 1 Division of Internal Medicine, Hospital de Clínicas de Porto Alegre, Porto Alegre, Brazil; 2 Graduate Program in Medical Sciences Endocrinology, Universidade Federal do Rio Grande do Sul, Porto Alegre, Brazil; 3 Department of Internal Medicine, Universidade Federal do Rio Grande do Sul, Rua Ramiro Barcelos 2350, Porto Alegre, 90035 003 Brazil; 4 Department of Physiology, Universidade Federal do Rio Grande do Sul, Porto Alegre, Brazil

**Keywords:** Fracture risk, Sarcopenia, Primary health care, Frailty, Older women, Osteoporosis

## Abstract

**Summary:**

Simple functional measures may help identify older women at high fracture risk. In this cross-sectional study, reduced handgrip strength, calf circumference, and lean mass were associated with higher risk of fracture by FRAX. These easily obtainable parameters may enhance fracture-risk screening in primary care.

**Purpose:**

To examine the associations between frailty, sarcopenia-related parameters, and fracture risk in community-dwelling older women, and to evaluate whether simple functional measures may help identify individuals at elevated risk of osteoporotic fractures.

**Methods:**

We conducted a cross-sectional study including women aged ≥ 60 years recruited from a primary care facility in Southern Brazil. Frailty was assessed using the Clinical Frailty Scale, and muscle strength and physical performance were evaluated using SARC-F, Timed Up and Go, gait speed, the Short Physical Performance Battery, calf circumference (CC), and handgrip strength (HGS). Bone mineral density and trabecular bone score (TBS) were measured by DXA. Ten-year fracture probability was estimated using the Brazilian FRAX® algorithm. Appendicular lean mass index (ALMI) and biochemical markers relevant to bone metabolism were also analyzed.

**Results:**

Among the 119 participants, 38.5% were classified as having high or very high 10-year fracture risk. Compared with women in the low-risk group, those at high risk were older and had lower BMI, TBS, HGS, CC, and ALMI, in addition to a higher comorbidity burden. In multivariable Poisson regression adjusted for age and comorbidity, lower muscle strength, reduced muscle mass, and poorer physical performance were independently associated with higher fracture risk prevalence, whereas frailty status was not. Among the muscle parameters, ALMI showed the strongest association with high fracture risk.

**Conclusion:**

Reduced muscle strength and lean mass were associated with higher fracture risk, even in women without established frailty or sarcopenia. HGS and CC emerge as simple, low-cost indicators that may enhance fracture-risk screening in primary care and complement conventional assessments such as DXA and FRAX.

## Introduction

The demographic shift towards an aging population is a global phenomenon, particularly pronounced in developing countries such as Brazil. This demographic trend is associated with an increased prevalence of age-related conditions including frailty, sarcopenia, and osteoporosis — all diagnoses that contribute significantly to morbidity and mortality in older adults [1].

Frailty is a multidimensional syndrome characterized by decreased physiological reserve and resistance to stressors, resulting in greater vulnerability to adverse outcomes such as falls, hospitalization, and death. Although often confused with sarcopenia, the latter specifically refers to the loss of skeletal muscle mass and function, which also contributes to disability and fracture risk [2].

Fractures due to osteoporosis are among the most debilitating consequences of aging, especially hip fractures, which are associated with high mortality and functional decline. Tools like Dual-energy X-ray Absorptiometry (DXA) and Trabecular Bone Score (TBS) assess bone density and quality, while FRAX estimates 10-year fracture risk [3].

Among the determinants of fracture risk, clinical frailty and sarcopenia emerge as relevant predictors due to their associations with reduced physical function, falls, and impaired bone quality. However, the interplay between these conditions and bone health remains underexplored in primary care contexts, especially in older women, where early detection may be most impactful. This study tries to fill that knowledge gap [4, 5].

This study aims to evaluate the association between clinical frailty and sarcopenia with the clinical risk of fractures calculated by FRAX in older women enrolled in primary care facilities (PCF). Specifically, we seek to assess frailty, sarcopenia and fracture risk in this population using validated tools and characterize clinical, functional, and densitometric profiles. In addition, we analyze serum biomarkers involved in bone metabolism—such as 25-hydroxyvitamin D, parathyroid hormone (PTH), calcium, phosphorus, albumin, and alkaline phosphatase—and explore their associations with fracture risk in the context. Furthermore, we seek to identify factors potentially serving as accessible screening tools in primary care settings.

## Methods

### Study design and participants

We conducted a cross-sectional observational study selecting participants from a population of older adults registered at PCF located in Porto Alegre, Brazil. Eligible participants were individuals aged 60 years or older residing under the coverage area of the health unit. Exclusion criteria included any condition that prevented the participant from attending the research center for in-person assessments.

The study protocol was structured as a multicenter design, enrolling participants across multiple research sites [6]. Assuming a 40% prevalence of low bone mass in sarcopenic participants, an estimated two-fold increased fracture risk in sarcopenic individuals compared to those without sarcopenia, and a power of 80% with a significance level of 5%, a minimum of 162 participants was required, according to estimates generated by the PSS Health online tool [7].

Participants were randomly selected from a registry of individuals meeting the eligibility criteria in the PCF database. Potential participants were contacted by telephone and invited to take part in the study. Those who agreed to participate provided written informed consent prior to completing the interview and physical assessments described below. Informed consent covered only the data collection procedures outlined in the approved study protocol.

The interview collected sociodemographic data regarding lifestyle habits (tobacco and alcohol use, physical activity, and dietary patterns), medical history, medication in use, previous falls, and hospitalizations. Frailty was assessed using the Clinical Frailty Scale [8]. Physical performance and strength were evaluated using SARC-F, Timed Up and Go test, 4-m gait speed, SPPB, and chair stand test according to EWGSOP2 [9]. Handgrip strength (HGS) was measured with a JAMAR dynamometer, following ASHT guidelines [10].

Calf circumference (CC) was measured with the participant standing upright, weight evenly distributed, and the calf muscle relaxed. A non-elastic measuring tape was placed horizontally around the point of maximal calf girth [11]. The Charlson Comorbidity Index (CCI) was used to assess the presence and severity of comorbid conditions, assigning weighted scores to 19 comorbidities based on their association with mortality risk [12].

Total body composition was analyzed using a standardized assessment by DXA using a Lunar Prodigy Primo device (Encore version 14.10; Radiation Corporation, Madison, WI, USA). Appendicular lean mass index (ALMI) was calculated as appendicular lean mass divided by height squared (kg/m^2^).

The same device assessed bone density, measured at the lumbar spine (L1–L4) and right total femur and femoral neck and expressed in g/cm^2^. In the presence of artifacts, the left femur was scanned. The non-dominant forearm was used as an alternative site when primary sites were unsuitable. Vertebral Fracture Assessment (VFA) scans were performed during DXA to check for vertebral fractures, which were graded according to Genant [13].

Bone mineral density (BMD) T-scores were determined using reference data from the NHANES III cohort. Participants were classified as having normal BMD (T-score ≥ –1.0), osteopenia (T-score between –1.0 and –2.5), or osteoporosis (T-score ≤ –2.5). Fracture risk was assessed using the FRAX algorithm, according to Brazilian version [14]. Trabecular Bone Score (TBS) was assessed using the TBS iNsight software, version 3.0.2.0 (Medimaps Group, Geneva, Switzerland), providing a microarchitectural evaluation for each participant in addition to conventional BMD measurements.

Additionally, serum levels of 25-hydroxyvitamin D, parathyroid hormone (PTH), phosphorus, alkaline phosphatase, total calcium, and albumin were measured through standardized laboratory procedures.

The present study protocol was approved by the Ethics Committee of Hospital de Clínicas de Porto Alegre (approval number 57139721.3.0000.5327). To ensure confidentiality, all data were anonymized prior to entry into the database and subsequent analysis.

### Statistical analysis

Data distribution was assessed using the Shapiro–Wilk test, and appropriate descriptive statistics were calculated for all variables. Continuous variables are presented as mean ± standard deviation when normally distributed, or as median (interquartile range) when non-normally distributed. Between-group comparisons were conducted using the independent samples t-test or the Mann–Whitney *U* test, as appropriate. Categorical variables were compared using the chi-square test. Poisson regression models with robust variance were performed to estimate prevalence ratios and corresponding 95% confidence intervals between groups.

Our primary outcome was to compare frailty- and sarcopenia-related tests between participants with high and low fracture risk. All analyses were conducted using IBM SPSS Statistics 18.0 (SPSS, Chicago, IL, USA) for Windows, and statistical differences were considered significant at *p* < 0.05.

## Results

**Fig. 1:**
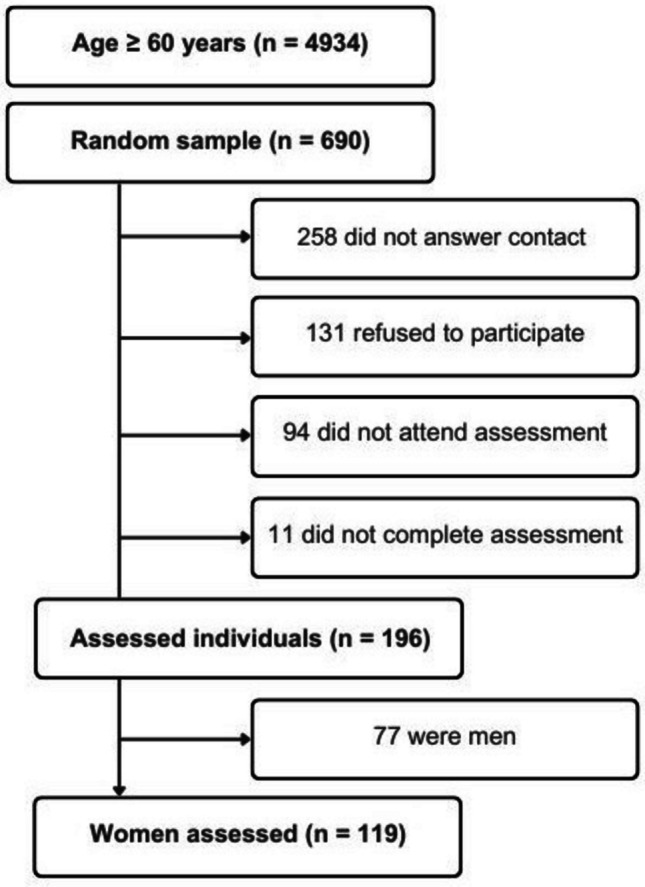
Participant selection flowchart

A database comprising 4934 community-dwelling individuals aged 60 years or older, of both sexes, was screened for eligibility. From this population, 690 individuals were randomly selected and invited to participate. Of these, 196 participants completed the full assessment protocol. After excluding men, the present analysis includes 119 women (Fig. 1).

Women who were not assessed did not differ from those evaluated in terms of skin color; however, they were older on average [median age 72 (66–80) vs. 68 (63–75) years; *p* < 0.001]. As no additional clinical or sociodemographic information was available for these women, a more comprehensive evaluation of their representativeness relative to the primary care population could not be performed.

Among the evaluated sample, 46 women (38.5%) were categorized as having a high or very high 10-year probability of sustaining a major osteoporotic fracture or a hip fracture, as estimated by FRAX. Comprehensive demographic and densitometric profiles of the study sample are presented in Table 1. Table 2 illustrates differences between groups regarding frailty and sarcopenia-related parameters.

**Table 1:** Demographic and densitometric characteristics of participants according to FRAX risk categories

Variable	Low FRAX	High FRAX	Total	*p*-value
Participants, *n* (%)	72 (61.0%)	46 (39.0%)	118 (100.0%)	
Age (years)	68 (64–74)	73 (65–79)	69 (65–76)	**0.029**
Body mass index (kg/m^2^)	29.2 ± 5.1	25.9 ± 4.3	27.9 ± 5.0	** < 0.001**
White skin color	55 (76.4%)	40 (88.9%)	95 (81.2%)	0.092
Current smoking	4 (5.6%)	5 (10.9%)	9 (7.6%)	0.309
Osteoporosis treatment	3 (4.2%)	10 (21.7%)	13 (11.0%)	**0.003**
Patients with moderate/severe fracture*	8 (14.3%)	8 (22.2%)	16 (17.4%)	0.327
Patients with falls in past year	20 (27.8%)	16 (34.8%)	36 (30.5%)	0.420
Right femoral neck T-score	− 0.9 ± 0.9	− 2.3 ± 0.7	− 1.4 ± 1.0	** < 0.001**
Right total hip T-score	− 0.2 ± 1.0	− 1.7 ± 1.3	− 0.8 ± 1.3	** < 0.001**
Lumbar spine L1–L4 T-score	0.1 ± 1.9	− 1.6 ± 1.2	− 0.5 ± 1.8	** < 0.001**
DXA-defined osteoporosis	8 (11.1%)	27 (58.7%)	35 (29.7%)	** < 0.001**
Trabecular bone score (TBS)	1.392 ± 0.096	1.308 ± 0.098	1.360 ± 0.105	** < 0.001**

Data are presented as mean ± SD, median (IQR), or *n* (%), as appropriate. Between-group comparisons were conducted using Student’s t-test, the Mann–Whitney *U* test, or the chi-square test, as appropriate. *P*-values < 0.05 are shown in bold. *as detected by vertebral fracture assessment

**Table 2:** Comorbidities, frailty status, and sarcopenia-related parameters of participants according to FRAX risk categories

Variable	Low FRAX	High FRAX	Total	*p*-value*
Participants, *n* (%)	72 (61.0%)	46 (39.0%)	118 (100.0%)	
Charlson comorbidity index	3 (2–4)	4 (3–5)	3 (3–4)	**0.001**
Clinical Frailty Scale	2 (2–3)	3 (2–4)	2 (2–3)	0.122
4‑m gait speed (m/s)	1.08 ± 0.26	1.07 ± 0.36	1.08 ± 0.30	0.900
Timed Up and Go (s)	10.0 (8.6–12.1)	10.5 (8.7–12.9)	10.3 (8.6–12.4)	0.158
SARC‑F score	1 (0–2)	2 (0–3)	1 (0–2)	0.058
SPPB	10 (8–11)	9 (8–10)	9 (8–11)	0.067
Handgrip strength (kgf)	22 (18–26)	19 (16–22)	20 (17–24)	**0.003**
Calf circumference (cm)	36.9 ± 3.8	34.2 ± 2.9	35.9 ± 3.7	** < 0.001**
ALMI (kg/m^2^)	7.1 ± 1.1	6.2 ± 0.8	6.8 ± 1.1	** < 0.001**

Data are presented as mean ± SD or median (IQR) as appropriate. *according to Student’s *t*-test or Mann–Whitney *U* test, as appropriate. *P*-values < 0.05 are shown in bold. *SPPB*, Short Physical Performance Battery; *ALMI*, Appendicular Lean Mass Index

Overall, the study sample demonstrated a largely preserved functional status: 83.0% of participants were classified as robust, 11.9% as pre-frail, and only 5.0% as frail according to CFS. Although the prevalence of densitometrical defined osteoporosis was high, the proportion of participants undergoing some kind of pharmacological treatment was low, only 34.2%. Notably, the number of participants with moderate or severe vertebral fractures were evenly distributed between fracture-risk groups. Analysis of laboratory parameters revealed no significant differences between groups, with the exception of serum creatine phosphokinase levels [102 (69–159) vs. 67 (51–110); *p* = 0.001].

In multivariable Poisson regression models with robust variance, adjusted for age and CCI, lower muscle strength, reduced muscle mass, and poorer physical performance were independently associated with a higher prevalence of high fracture risk as calculated by FRAX. Notably, ALMI differed significantly between groups even in the absence of an established diagnosis of sarcopenia and demonstrated the strongest association with high fracture risk, whereas frailty status was not significantly associated. Detailed effect estimates are presented in Table 3.

**Table 3:** Association between functional and muscular variables and high fracture risk categorization by FRAX

Variable	Unit of increase	Adjusted PR (95% CI)	*p*-value
Clinical Frailty Scale	+ 1 point	1.08 (0.88–1.33)	0.477
4‑m gait speed (m/s)	+ 0.1 m/s	1.06 (0.97–1.16)	0.190
Timed Up and Go (s)	+ 1 s	1.03 (1.00–1.07)	**0.036**
SARC‑F score	+ 1 point	1.09 (0.98–1.21)	0.111
SPPB	+ 1 point	0.92 (0.85–1.00)	**0.040**
Handgrip strength (kgf)	+ 1 kgf	0.95 (0.91–0.99)	**0.020**
Calf circumference (cm)	+ 1 cm	0.90 (0.84–0.96)	**0.002**
ALMI (kg/m^2^)	+ 1 kg/m^2^	0.62 (0.51–0.77)	** < 0.001**

Adjusted prevalence ratios (PR) and 95% confidence intervals (95% CI) were obtained from Poisson regression models with robust variance estimation, controlling for age and comorbidity burden (Charlson Comorbidity Index). *P*-values < 0.05 are shown in bold

The overall prevalence of frailty and sarcopenia in the sample was very low (5% and 3.4%, respectively), limiting the ability to perform between-group comparisons. Based on the observed sample size and group distribution, the estimated statistical power to detect differences between high and low FRAX groups was approximately 85% for handgrip strength, 95% for calf circumference, and 99% for ALMI, corresponding to Cohen’s d effect sizes of 0.56, 0.76, and 0.92, respectively.

## Discussion

In this cross-sectional study of older women from primary health care, we identified a substantial proportion of women with high or very high fracture risk as calculated by FRAX, despite a low prevalence of sarcopenia and overall preserved functional status.

The prevalence of densitometric osteoporosis was comparable to that reported in similar community-based cohorts in Latin America [15, 16]. However, the low rate of pharmacological treatment highlights a persistent treatment gap, consistent with global evidence of underdiagnosis and undertreatment of osteoporosis, particularly in primary care settings [17]. These findings reinforce the importance of systematic fracture risk assessment in this context.

Moderate and severe vertebral fractures were evenly distributed across risk groups, underscoring the often silent nature of vertebral fractures and suggesting that screening strategies may be justified even among apparently low-risk individuals. Although FRAX is a valuable clinical tool, its limitations when used in isolation have been previously described [18].

Muscle-related parameters (HGS, CC, ALMI) seemed to be inversely associated with the presence of high fracture risk by FRAX, even in our highly functional sample. These findings support the concept that reduced muscle strength and lower lean mass reflect increased skeletal vulnerability. HGS, in particular, has been previously identified as a surrogate marker of global muscle strength and a predictor of falls, disability, and fractures [19–21]. Notably, the low rates of frailty and sarcopenia in our sample suggest that fracture risk may be increased even before overt muscle dysfunction or frailty become clinically apparent. This suggests their potential utility as accessible, low-cost screening tools in primary care.

In contrast, likely reflecting the overall high functional status and preserved mobility of our sample, mobility-based tests may have exhibited limited variability, thereby reducing their ability to detect significant differences between groups. This interpretation is consistent with emerging evidence suggesting that deficits in muscle strength, rather than mobility impairment alone, may be more closely related to skeletal fragility [22, 23].

Biochemical markers were largely similar between risk groups, except for creatine phosphokinase (CPK), and its relevance in fracture risk stratification remains uncertain and warrants further investigation [24]. The absence of differences in vitamin D, PTH, calcium, and phosphorus levels may reflect the clinical homogeneity and preserved functionality of the cohort.

This study has several limitations. Its cross-sectional design precludes causal inferences, and potential selection bias may have limited the representativeness of the primary care population. The relatively small final sample size may have reduced statistical power, particularly for mobility-related variables. Furthermore, the reliance on self-reported comorbidities and lifestyle factors may have introduced reporting bias and residual misclassification. The relatively preserved functional status of the cohort likely contributed to reduced variability in clinical parameters, potentially attenuating certain associations. Relationships between muscle, function, and fracture risk may be more pronounced in populations with greater heterogeneity and functional impairment.

Despite these limitations, the study has notable strengths, including its primary care–based sample and the comprehensive assessment of muscle and functional parameters using standardized measures. These features enhance the clinical relevance of the findings. Well-designed prospective studies are warranted to determine the incremental predictive value of muscle mass, strength, and functional performance when incorporated into established fracture risk prediction models.

Overall, our findings support the concept of osteosarcopenia, a condition characterized by the coexistence of reduced bone mass and impaired muscle parameters, increasingly recognized as a high-risk phenotype in older adults. Importantly, the associations observed in this study suggest that subtle declines in muscle strength and body composition may already signal increased fracture risk. These results highlight the close interplay between bone and muscle health and suggest that muscular parameters may serve as early indicators of skeletal fragility.

## Conclusion

Our findings suggest an association between functional parameters and FRAX-calculated fracture risk in older women assessed in primary care, even among individuals with relatively preserved functional status. However, as this study did not formally incorporate these parameters into predictive fracture models, future longitudinal studies are needed to determine whether their inclusion may provide incremental value in fracture risk assessment and contribute to improve clinical outcomes in aging populations.

## Data Availability

The datasets generated and/or analyzed during the current study are available from the corresponding author on reasonable request.
